# Improving Soil Properties and Microbiomes by Mixed *Eucalyptus–Cupressus* Afforestation

**DOI:** 10.3390/biology14121667

**Published:** 2025-11-24

**Authors:** You-Wei Zuo, Yu-Ying Liu, Ya-Xin Jiang, Wen-Qiao Li, Yang Peng, Sheng-Mao Zhou, Shi-Qi You, Sheng-Qiao Liu, Hong-Ping Deng

**Affiliations:** 1 Center for Biodiversity Conservation and Utilization, Key Laboratory of Eco-Environment in the Three Gorges Reservoir Region, Ministry of Education, School of Life Sciences, Southwest University, Beibei, Chongqing 400715, China; 2 Chongqing Key Laboratory of Plant Resource Conservation and Germplasm Innovation, Institute of Resources Botany, School of Life Sciences, Southwest University, Beibei, Chongqing 400715, China; 3 College of Pharmaceutical Sciences, Southwest University, Beibei, Chongqing 400715, China

**Keywords:** comprehensive evaluation, ecological restoration, mixed forests, plant–soil–microbial interactions, plant and soil properties

## Abstract

Monoculture plantations of *Eucalyptus* in China have raised ecological concerns due to water depletion, soil degradation, and fire risk. Integrating *Eucalyptus* with *Cupressus* offers a sustainable approach to improving forest ecosystem health. In this study, we established five forest treatments, pure *Eucalyptus* (1:0), mixed *Eucalyptus*–*Cupressus* at three ratios (2:1, 1:1, and 1:2), and pure *Cupressus* (0:1), to assess their effects on soil properties, microbial diversity, and metabolomic profiles. Laboratory analyses revealed significant differences in physicochemical soil properties (such as water content (*p* < 0.05), pH (*p* < 0.001), organic carbon (*p* < 0.001), and nitrogen (*p* < 0.001)) among various groups within the mixed forests. Microbial community investigations highlighted a unique microbial signature in *Eucalyptus*–*Cupressus* mixed forests, especially when the tree ratio was 1:2, characterized by a rich (Chao1, *p* < 0.05) and diverse (Shannon, *p* < 0.05) array of bacterial taxa. The mixed *Eucalyptus*–*Cupressus* forest also exhibited an uplift in microbial communities, bacterial genera such as *RB41*, and fungal genera including *Penicillium*, *Talaromyces*, and *Mortierella*, which are associated with enhanced organic matter decomposition and nutrient cycling. Interactive networks within microbial communities were revealed through co-occurrence and Spearman correlation analyses, highlighting potential symbiotic relationships and ecological complexities. Metabolomic analysis, coupled with pathway analysis, further illuminated metabolic shifts in the mixed forests, emphasizing alterations in key metabolic pathways such as phenylpropanoid biosynthesis, tyrosine metabolism, arachidonic acid metabolism, and isoquinoline alkaloid biosynthesis. Collectively, these results show that moderately mixed *Eucalyptus*–*Cupressus* forests improve soil fertility and microbial multifunctionality, providing a practical model for sustainable and resilient forest management in subtropical regions.

## Simple Summary

Forests are important ecosystems that provide many benefits to the environment and human society, such as clean air, water regulation, and carbon storage. However, different types of forests, such as natural forests, plantations, and mixed forests, may affect the soil and its biological communities in different ways. In this study, we explored how various forest types in a subtropical mountain area influenced the soil’s physical and chemical properties, microbial communities, and chemical compounds known as metabolites. We found that soil in mixed forests had higher levels of nutrients and supported a more diverse and beneficial group of microbes compared to monoculture plantations. These microbes were also linked with compounds that are important for nutrient cycling and plant health. By using advanced analysis techniques, we were able to understand how changes in forest type can impact the soil’s ability to support healthy ecosystems. This research is valuable for forest managers, conservationists, and policymakers who aim to improve forest quality and sustainability. It shows that choosing more diverse forest types can promote healthier soil, which benefits the entire ecosystem and the services it provides to people.

## 1. Introduction

*Eucalyptus* trees (e.g., *E. grandis* and *E. urophglla*) are well-suited for cultivation in areas with abundant sunlight, such as plains, slopes, and roadsides [1]. These trees exhibit strong adaptability to soil conditions, possess well-developed root systems, fast growth, a short maturation period, and have wide applications in industries like construction and paper production [2]. Due to its distinctive biological characteristics, many countries and regions have introduced *Eucalyptus* for afforestation and timber production [3,4]. However, with the extensive and large-scale planting of *Eucalyptus*, ecological issues in *Eucalyptus* plantations have become increasingly prominent. Growing evidence has raised concerns that the extensive root system of *Eucalyptus*, which depletes surface and groundwater during its rapid growth, leading to rapid water evaporation and causing soil aridity and compaction in forested areas [5]. *Eucalyptus* is believed to deplete soil fertility significantly due to its rapid growth, leading to severe soil degradation, which subsequently hinders the growth of subsequent generations of *Eucalyptus* or alternative tree species [6]. Its high-water consumption leads to soil aridity and nutrient depletion, adversely affecting the growth of native plants [4]. Moreover, the allelopathic properties of *Eucalyptus* release toxins into the soil, inhibiting the germination and growth of other plant species and further exacerbating soil toxicity [7]. The dense root systems of *Eucalyptus* trees also compact the soil, reducing its porosity and disrupting the natural soil structure, which diminishes its ability to retain water and support diverse microbial communities. Furthermore, *Eucalyptus* branches, leaves, and trunks contain a significant amount of oil. In the continuous high temperatures of summer, they produce a highly flammable eucalyptus oil, and transform *Eucalyptus* forests into potential “gasoline bombs” [8,9]. It has been reported that the total *Eucalyptus* cultivation area in China spans 5,460,000 hectares, with a dominant presence in southwestern China, particularly in Sichuan and Chongqing Provinces [10,11]. This extensive cultivation of *Eucalyptus* highlights the urgency of exploring alternative strategies to address the challenges arising from the high-density planting of *Eucalyptus* and to establish a sustainable forest ecosystem.

Mixed forests of *Eucalyptus*, in combination with tree species like *Quercus* spp., *Parashorea* spp., *Acacia* spp., *Manglietia* spp., *Pinus* spp., *Erythrophleum* spp., and *Castanopsis* spp., have demonstrated substantial economic and ecological benefits compared to monoculture *Eucalyptus* forests [12,13]. These mixed forests are able to foster the healthy growth of *Eucalyptus* and improve the physicochemical properties of the forest soil in their early stages. Additionally, *Cupressus* spp. thrives in warm and humid conditions and exhibits adaptability to various soil types. When combined with slow-growing nitrogen-fixing tree species, *Cupressus* spp., such as *C. funebris*, contributes to the formation of robust artificial forest communities, making it suitable for afforestation in hilly and mountainous regions with poor soil quality [14]. *Eucalyptus*–*Cupressus* mixed forests have demonstrated advantages in terms of understory biodiversity and leaf litter composition compared to pure *Eucalyptus* and *Cupressus* forests [15,16], whereas the detailed information about soil nutrients cycling and microbial structures in the mixed forest ecosystems remains elusive.

Within the intricate microenvironment of forest soil, soil metabolites serve as crucial connectors linking soil, plants, and soil microorganisms. Secondary metabolites produced by plants are released into the soil through leaching and volatilization from aboveground plant parts. These compounds influence the growth and development of surrounding plants, soil organisms, and microorganisms [17,18]. In turn, soil microbial activity can affect nutrient absorption in plants and the synthesis and secretion of plant metabolites. It has been documented that soil microbiota can assist plants in mitigating abiotic stress and activating nutrient cycling in different mixed forest ecosystems [19]. Some metabolites produced by soil microorganisms play a critical role in regulating various biological activities of neighboring organisms. For instance, certain nitrogen-fixing bacteria may form associations with the roots of specific tree species in the mixed forest, leading to increased nitrogen inputs through biological nitrogen fixation [20]. The fixed nitrogen becomes available to all trees, such as *Eucalyptus* and *Cupressus* trees, helping to mitigate abiotic stress, particularly in nitrogen-deficient environments. However, the intricate relationships and interactions between various tree species, especially *Eucalyptus*–*Cupressus* mixed forests, and the associated soil microbiota remain an area of research that requires further exploration.

This study hypothesizes that mixed forests of *Eucalyptus* and *Cupressus* can significantly enhance soil physicochemical properties, microbial community composition, and soil metabolite profiles compared to monoculture systems. We further anticipate that different mixing ratios of these two species will lead to distinct shifts in microbial structure and metabolic expression patterns, reflecting complex ecological interactions. The objective of this research is to elucidate the tree–soil–microbe–metabolite interplay under varying plantation compositions, thereby providing a mechanistic understanding of mixed forest ecosystem functioning. By integrating multi-omics approaches with soil chemical analyses, this study aims to generate novel insights that will inform ecological restoration practices and support the development of more sustainable and resilient forest ecosystems, particularly in regions impacted by large-scale *Eucalyptus* afforestation.

## 2. Materials and Methods

### 2.1. Site Description

The research area is situated in the Yubei District of Chongqing, China, and experiences a pronounced subtropical humid climate characterized by distinct continental monsoon patterns. The region has an average temperature of 17.3 °C, and the annual precipitation averages around 1100 mm. Native vegetation includes subtropical evergreen and deciduous broad-leaved forests, mixed coniferous and broad-leaved forests, and evergreen broad-leaved forests, complemented by extensive artificial forest plantations.

The research area includes forest plots with similar site conditions and approximately 15-year-old *Eucalyptus* (*E. grandis* × *E. urophglla*) and *Cupressus* (*C. funebris*) artificial mixed forests or pure forests. Historically, the area was composed of anthropogenically influenced mixed woodlands, where early-stage plantations of *Eucalyptus* were interspersed with native shrubs and grasses; however, long-term dominance and allelopathic interference from *Eucalyptus* eventually transformed much of the landscape into monoculture eucalyptus forests. Five afforestation patterns were established, with each denoted as follows: *Eucalyptus* pure forest (named as group A, *Eucalyptus*–*Cupressus* 1:0), *Eucalyptus* and *Cupressus* mixed forests with tree ratios of 2:1 (group B), 1:1 (group C), 1:2 (group D), and *Cupressus* pure forest (group E, *Eucalyptus*–*Cupressus* 0:1). In 2021, 20 m × 20 m sample plots were randomly established within each type of forest plot. The individual trees within these sample plots were examined, and data on tree height, diameter at breast height (DBH), and other relevant measurements were collected and recorded (Table S1).

### 2.2. Soil Sampling

Soil samples were collected from each forest type using a serpentine five-point sampling method within each 20 m × 20 m plot. After carefully removing surface litter and fallen branches, bulk soil was sampled from the top 0–20 cm layer using a sterile auger at each point. In total, three biological replicates were collected for each forest treatment. Subsamples from each of the five points within a plot were thoroughly mixed to form one composite soil sample per replicate. Each composite sample was then divided into two portions. One portion was immediately weighed and oven-dried at 105 °C to determine soil water content. The other portion was transferred into sterile, self-sealing plastic bags, stored on ice in the field, and transported to the laboratory. Upon arrival, the samples were passed through a 2 mm sieve to remove debris and roots, and the processed soils were stored at −80 °C for subsequent physicochemical, microbial, and metabolomic analyses.

### 2.3. Soil Physical and Chemical Properties

A 10 g soil sample was sieved through a 1 mm mesh and combined with 25 mL of distilled water. After standing for 30 min, the suspension pH was measured using a PH meter (PHS-2F). For soil organic carbon (OC) measurement, 0.1–0.5 g (exact value recorded) of soil sample was sieved through a 60-mesh sieve (<0.25 mm) and then mixed with 10 mL of 0.36 mol/L potassium dichromate-sulfuric acid solution. The mixture was thoroughly shaken and boiled at 185–190 °C. After cooling, 3–4 drops of phenanthroline were added to the solution, followed by the addition of a standard solution of ferrous sulfate (FeSO_4_) (0.2 mol/L). The content of the added FeSO_4_ was recorded, and the soil organic matter was subsequently calculated using the previously established method. For soil water content (WC) detection, the fresh soil sample was precisely weighed and then subjected to a 12 h baking process in a preheated oven at 105 °C. Afterward, the treated soil sample was transferred to a dryer and allowed to cool to room temperature for 30 min before being weighed again. The calculation method used was consistent with the previous approach. Soil total nitrogen (TN) was analyzed using the semimicro-Kjeldahl (KDY-9820) digestion method. Soil total phosphorus (TP) was quantified colorimetrically using the molybdate method. Soil total potassium (TK) was measured through flame spectrophotometry. Soil available nitrogen (AN) was determined using the potassium dichromate external heating method. Soil available phosphorus (AP) was analyzed using the molybdenum blue method. Soil available potassium (AK) was extracted using 1 M ammonium acetate (pH 7.0) and quantified via flame photometry.

### 2.4. DNA Extraction and High-Throughput Sequencing

Initially, we evaluated soil microbiome composition and diversity of three main mixed types—*Eucalypt–Cypress* mixed forest, *Eucalypt–Ficus* mixed forest and *Eucalypt–Ginkgo* mixed forest—and *Eucalypt* pure forest. Soil DNA extraction from these four groups and different ratio of *Eucalyptus*–*Cupressus* mixed forests was conducted, with 0.5 g of soil utilized for each of the replicates. The extraction process was carried out using the PowerSoil DNA Isolation Kit, following the manufacturer’s instructions. To ensure the purity and quality of the extracted DNA, a combination of PCR amplification and subsequent 2% agarose gel electrophoresis was employed. Specific primers were chosen for the amplification of soil bacterial and fungal structures. The bacterial 16S rDNA (V3 + V4) region was targeted by the primers 338F and 806R, while the fungal ITS sequences were amplified using the primers ITS1F and ITS2R. Each 25 µL PCR reaction contained 12.5 µL of 2× Taq PCR Master Mix (Takara, Japan), 1 µL of each primer (10 µM), 1 µL of DNA template (~10 ng), and 9.5 µL of nuclease-free water. The thermal cycling program consisted of an initial denaturation at 95 °C for 3 min; 30 cycles of 95 °C for 30 s, 55 °C for 30 s, and 72 °C for 45 s; and a final extension at 72 °C for 10 min. PCR products were verified by 2% agarose gel electrophoresis, and clear amplicons were purified using the GeneJET Gel Extraction Kit (Thermo Scientific, Waltham, MA, USA). A secondary 8-cycle PCR was performed to attach Illumina sequencing adapters and indices. PCR products displaying distinct bands were then subjected to purification using the GeneJET gel extraction kit, following the guidelines provided by Thermo Scientific. Subsequently, equal concentrations of PCR products from each sample were prepared for sequencing on the Illumina MiSeq platform, employing 300-bp paired-end reads. This sequencing was carried out at TinyGene Bio-Tech Co., Ltd. in Shanghai, China, using technology provided by Illumina, Inc., based in San Diego, CA, USA.

### 2.5. Sequence Processing and Bioinformatic Analysis

To assess the microbial species composition in each sample site, the effective data from all soil samples were clustered into Operational Taxonomic Units (OTUs) at a 97% identity threshold. For bacterial 16S rRNA gene sequences, the Greengenes database was used (v2022.10, https://ngdc.cncb.ac.cn/databasecommons, accessed on 18 November 2025), and for fungal ITS sequences, the UNITE database was employed (v10.0, https://unite.ut.ee/), with species taxonomy annotation performed using the classify-sklearn algorithm. Alpha diversity of microbial communities was comprehensively assessed using QIIME2 (2019.4) software. Alpha diversity differences between different mixed forest modes were visually presented through graph generation with Kruskal–Wallis rank-sum tests and Dunn’s post hoc tests to validate the significance of differences. Utilizing QIIME2, a distance matrix was computed for differential OTU tables, followed by principal coordinates analysis (PCoA) analysis. Anosim and Permdisp algorithms were used to test the significance of differences among groups. The distance matrices were analyzed and visualized using R scripts, the Vegan package, and Ggplot2 package. Venn diagrams and heatmaps for microbial composition analysis were created using R scripts, the VennDiagram package (v1.7.3), and the Pheatmap package (v1.0.13). A co-occurrence network among different microbial taxa was constructed based on Spearman correlation analysis, with multiple hypothesis testing controlled using the Benjamini–Hochberg procedure to adjust for false discovery rate (FDR); only taxa pairs showing strong positive correlations (correlation coefficient >0.8) and adjusted *p*-values (FDR) below 0.01 were retained for network visualization. This network was created utilizing the “igraph” (v2.2.1) and “Hmisc” (v5.2.4) packages, which utilized unique correlations derived from pairwise comparisons of OTU abundance. To minimize potential bias from sequencing depth or sample sparsity, network stability and randomization tests were performed using permutation-based null models to differentiate true biological interactions from spurious correlations. To describe the network structure, various network topology metrics and features, such as modularity and eigenvector centrality, were applied. The network was then visualized using Gephi software (v0.10.1) with the Fruchterman Reingold layout. Linear discriminant analysis (LDA) was conducted using the R package “lefser” (v3.22) to identify microbial taxa that significantly differentiated the mixed forest types, thereby revealing potential biomarkers associated with variations in microbial community composition. Functional analysis and significance (*p*-value < 0.05) were conducted and presented using the “tidyverse” (v2.0.0) and “ggplot2” (v4.0.1) packages. Functional predictions for 16S rRNA and ITS sequences were conducted using PICRUSt2, and differential metabolites underwent functional pathway enrichment and topological analysis using the MetaboAnalyst (v4.0) package. Pathway enrichment results were visualized using KEGG Mapper (v5.1). Spearman rank correlation analysis was used to explore the associations between microbial communities and soil characteristics. Additionally, we employed structural equation modeling (SEM) to explore the relationships between soil properties (e.g., water content, organic matter, pH, and nutrients), microbial properties (such as gene abundance, diversity, and network complexity), and soil metabolomic multifunctionality. To evaluate model fit, we used various parameters including the chi-square (χ^2^) test, *p*-value (>0.05), the root mean square error of approximation (RMSEA) ranging from 0 to 0.05, and a high goodness-of-fit index (GFI) exceeding 0.90. This analysis was conducted using Amos version 25.0.

### 2.6. Metabolome Sequencing and Differentially Expressed Metabolites (DEMs) Analysis

Soil samples from every group were used for metabolite extraction. To each homogenized sample, we added 500 mL of 80% methanol and vortexed them for 5 min. Subsequently, each sample was partitioned into 20 mL portions to ensure quality. Afterward, all the solutions underwent centrifugation at 15,000× *g* for 20 min at 4 °C. Following this, the supernatant was diluted with water and once more subjected to centrifugation at 15,000× *g* for 20 min at 4 °C. The resulting supernatant was utilized for the LC-MS analysis. For liquid chromatography and mass spectrometry, we employed the Thermo Ultimate 3000 system and Thermo Q Exactive mass spectrometer, respectively (Thermo Scientific, Waltham, MA, USA). To ensure data quality, pooled quality control (QC) samples were prepared by mixing equal aliquots from all experimental samples and injected regularly throughout the run to monitor instrument stability and signal drift. Blank samples were also included to detect background noise or contamination. We executed dynamic exclusion to eliminate redundant data in the metabolome dataset. After standardizing the quantitative outcomes, we acquired metabolite identification and relative quantitative data. Utilizing the “XCMS” (v4.0) package in R, we performed the identification, filtration, and alignment of the produced MS/MS peaks. The obtained metabolites were annotated using the KEGG, HMDB, and LIPIDMaps databases. After standardization, partial least squares-discriminant analysis (PLS-DA) was conducted with the “ropls” package to determine the differentially expressed metabolites (DEMs) in the comparisons. DEMs were identified based on the threshold of variable importance in the projection (VIP values ≥ 1) and a *p*-value of ≤0.05. Redundancy Analysis (RDA) was conducted using the vegan (v2.5-7) package in R to assess the relationship between microbial communities and metabolite profiles, with significance tested via permutation.

## 3. Results

### 3.1. Soil Properties in Different Mixed Forests

**Figure 1:**
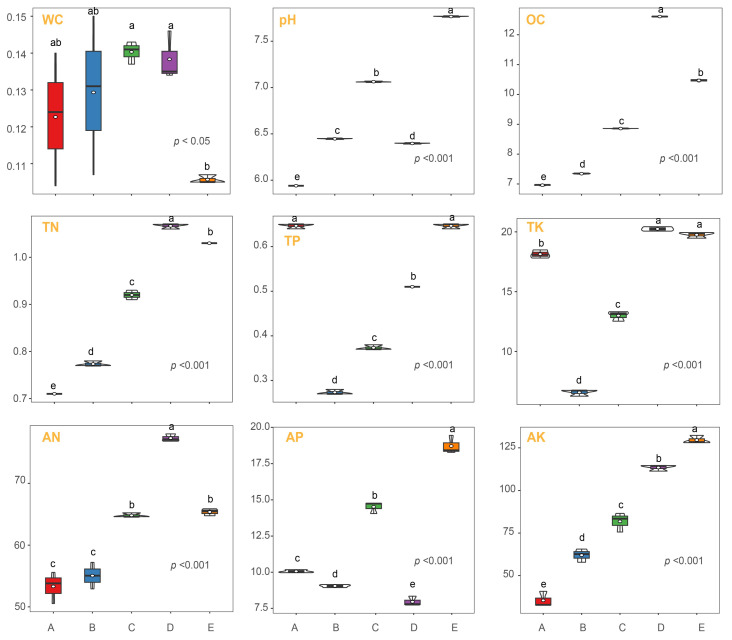
Box plot demonstrating soil property variations across distinct mixed forests. Key soil parameters measured include water content (WC), pH, organic carbon (OC), total nitrogen (TN), total phosphorus (TP), total potassium (TK), available nitrogen (AN), available phosphorus (AP), and available potassium (AK). The depicted forests range from *Eucalyptus* pure forests (group A, *Eucalyptus*–*Cupressus* 1:0), *Eucalyptus* and *Cupressus* mixed forests in various proportions (groups B, C, D), to *Cupressus* pure forests (group E, *Eucalyptus*–*Cupressus* 0:1). Different lowercase letters above the boxes indicate significant differences (*p* < 0.05) between groups based on Kruskal–Wallis test followed by Dunn’s post hoc comparisons.

Soil WC exhibited significant differences among the various mixed forests (*p* < 0.05) (Figure 1). Compared to groups A, B and E, mixed forests C and D had notably higher soil WC. Soil pH and OC content also showed significant variations among the different mixed forests (*p* < 0.05). Soil pH was highest in group E, followed by C, B, D, and A. OC content was highest in group D, followed by E, C, B, and A. Soil TN, and AN had the highest content in group D. Soil TK presented the highest in groups D and E. TP was highest in groups A and E, while AP was highest in group E. Group E had the highest AK content, while group A had the lowest.

### 3.2. Microbial Community Composition in Different Mixed Forests

Initially, we evaluated the soil microbiome composition and diversity of four forest types: *Eucalypt–Cypress* mixed forest, *Eucalypt–Ficus* mixed forest, *Eucalypt–Ginkgo* mixed forest, and pure *Eucalypt* forest. The results showed that the *Eucalypt–Cypress* mixed forest had higher bacterial and fungal composition and diversity indexes compared to the other three groups (Table S2 and Figure S1). Consequently, we focused our analysis on the *Eucalypt–Cypress* mixed forest with different tree ratios.

**Figure 2:**
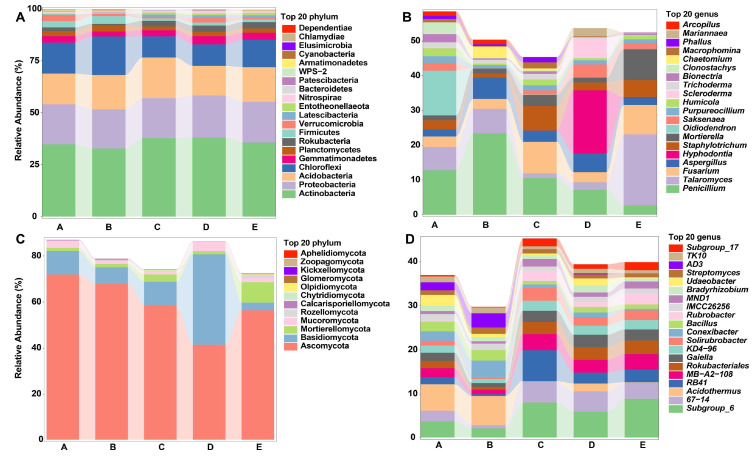
Microbial community taxonomic distribution depicted through bar plots at phylum (**A**,**C**) and genus (**B**,**D**) levels for bacterial and fungal community compositions. The microbial communities are ranked based on their relative abundance across all replicates, showing a decreasing order of prevalence.

The rarefaction curves for bacterial and fungal communities in different soil samples reached a plateau (Figure S2), indicating that the actual bacterial and fungal community compositions in the soil samples were reliably reflected by the sequencing data. The five types of mixed *Eucalyptus* and *Cupressus* forests revealed their affiliations with 33 phyla, 105 classes, 242 orders, 409 families, 777 genera, and 2013 species. Venn diagrams illustrated the unique and shared OTUs among the sample groups, showing significant differences in the number of shared OTUs among the samples (Figure S3). At the phylum level, the dominant bacterial phyla in different forest soils were Actinobacteria, Proteobacteria, Acidobacteria, and Chloroflexi, accounting for approximately 75% of the total abundance (Figure 2A). The relative abundance of Chloroflexi in group C was lower than in other groups, while the relative abundance of Acidobacteria was higher. At the genus level, the dominant bacterial genera in different forest soils included *Acidothermus*, *Rokubacteriales*, and *Gaiella* (Figure 2B). *Acidothermus* had a higher relative abundance in soil samples from groups A and B, while some groups (e.g., *subgroup_6*, *RB41*, and *67-14*) had higher relative abundances in groups C, D, and E.

For fungi, all annotated fungal OTUs belonged to 15 phyla, 43 classes, 107 orders, 255 families, 475 genera, and 736 species. Group A had the highest number of fungal species, with 420 species, followed by group C with 348 species. Venn diagrams showed that group C had more shared and unique OTUs than the other groups, whereas groups B and E had fewer shared and unique OTUs (Figure S3). At the phylum level, the dominant fungal phyla included Ascomycota, Basidiomycota, Mortierellomycota, and Mucoromycota, constituting approximately 80% of the total abundance (Figure 2C). The Ascomycota fungal phylum in group D had a lower relative abundance compared to other groups, while the Basidiomycota fungal phylum showed a significantly higher relative abundance. At the genus level, dominant fungal genera in different forest soils were *Penicillium*, *Talaromyces*, *Fusarium*, *Aspergillus*, *Hyphodontia*, *Staphylotrichum*, and *Mortieralla*, representing about 30% of the total abundance (Figure 2D).

### 3.3. Soil Microbial Diversity in Different Mixed Forests

**Figure 3:**
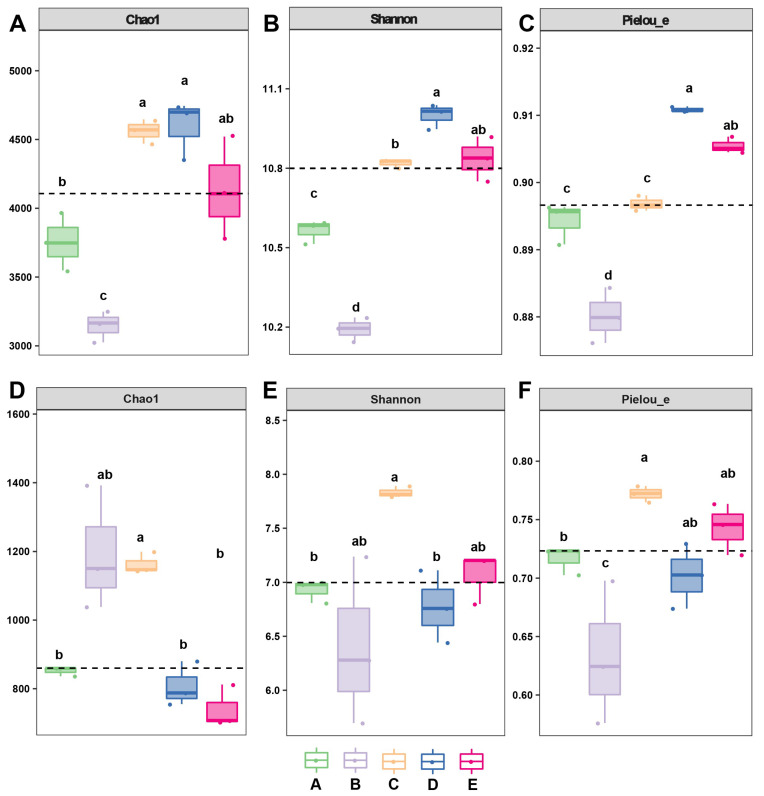
Alpha diversity indices of community groups, showcasing general bacterial (**A**–**C**) and fungal (**D**–**F**) alpha-diversity patterns through Chao1, Shannon, and Pielou indices across distinct mixed forests. Different lowercase letters above the boxes indicate significant differences (*p* < 0.05) between groups based on Kruskal–Wallis test followed by Dunn’s post hoc comparisons. The dashed line represents the overall mean value of each diversity index across all groups, serving as a reference baseline for comparison.

Significant differences were observed in the α-diversity indices among the soil samples from different groups. For bacterial communities, the richness indexes (Chao1, Shannon, and Pielou) were the highest in group D (Figure 3A–C). Adonis analysis demonstrated that there was a highly significant difference (*p* < 0.001) in bacterial community composition among the five sample groups. Interestingly, for fungal communities, the richness index (Chao1) ranked as A > C > B > D > E, the Shannon diversity index ranked as C > E > B > D > A, and the Pielou evenness index ranked as C > E > B > D > A (Figure 3D–F). Adonis analysis also revealed a highly significant difference (*p* < 0.001) in fungal community composition among the five sample groups.

### 3.4. Co-Occurrence Network Analyses of Bacteria and Fungi

**Figure 4:**
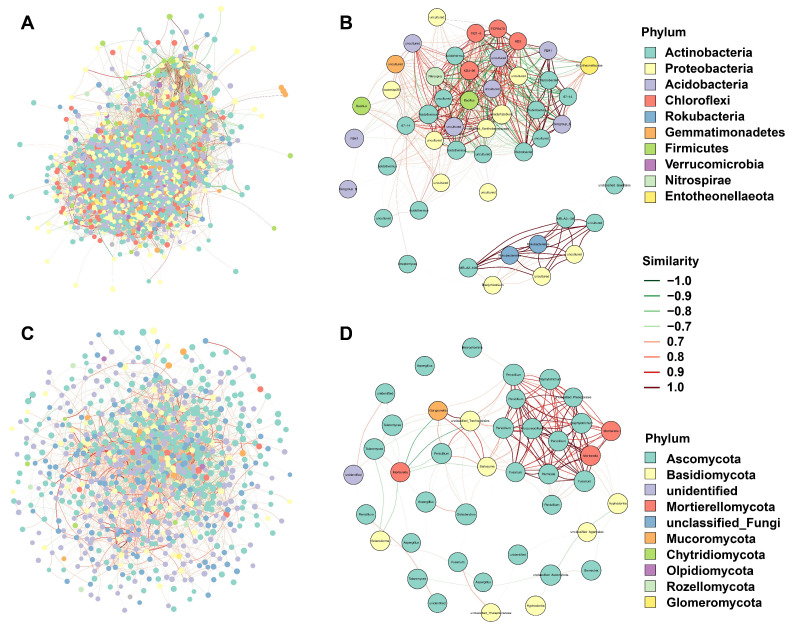
Network diagrams illustrating the modular association of bacteria (**A**,**B**) and fungi (**C**,**D**), along with a subnetwork of dominant species. Nodes represent OTUs in the groups, and node size is proportional to their abundance (log2(CPM/n). The top 10 modules are color-coded, with node size reflecting their abundance. Edges between nodes indicate correlations, with red lines denoting positive correlations and green lines denoting negative correlations.

In this study, network analysis was employed to investigate the co-occurrence patterns within the soil bacterial and fungal communities. For bacteria, the modularity index was 0.56, and the network density was 0.059 (Figure 4A). Notably, the top four taxa, namely *Acidothermus*, *Bacillus*, *Rubrobacter*, and *Entotheonellaceae*, were identified as hub biomarkers due to their high Betweenness scores. Additionally, based on node abundance, a dominant species sub-network was constructed by extracting the top 50 nodes in terms of average abundance (Table S3). In this sub-network, the major dominant nodes were Actinobacteria and Proteobacteria (Figure 4B). For fungi, the network consisted of 44 key nodes, with a modularity index of 0.61 and a network density of 0.023 (Figure 4C). Among these, the top four taxa, *Penicillium*, *Saitozyma*, *Humicola*, and *Fusarium*, were identified as hub biomarkers due to their high Betweenness scores (Figure 4D). In the dominant species sub-network, the primary dominant nodes were Ascomycota and Basidiomycota.

### 3.5. Microbial Biomarkers and Functional Analysis in Different Mixed Forests

**Figure 5:**
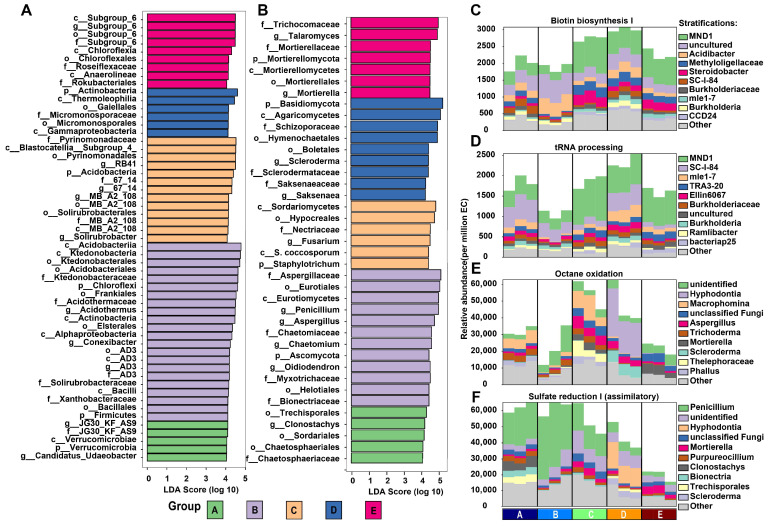
Differential composition and functionality of bacterial and fungal communities within varied forest groups. (**A**,**B**) LDA highlighting distinct variances in bacterial and fungal taxa across five mixed forests. (**C**–**F**) Display of bacterial and fungal community functionalities via the MetaCyc pipeline for microbial function annotation.

LDA was employed to uncover distinctive variations in bacterial and fungal taxa. The results unveiled that the highest number of bacterial and fungal taxa were observed in the D group (LDA scores > 4), while the E group exhibited a relatively lower number of taxa. Specifically, the D group featured three bacterial taxa, namely *Acidothermus*, *Conexibacter*, and *AD3* (Figure 5A), alongside four fungal taxa including *Penicillium*, *Aspergillus*, *Chaetomium*, and *Oidiodendron* (Figure 5B). On the other hand, the C group contained four distinctive bacterial taxa (*RB41*, *67*_*14*, *MB*_*A2*_*108*, and *Solirubrobacter*), along with one fungal taxon, *Fusarium*. Subsequently, functional annotation was carried out to assess potential functional disparities among the different sites (Figure 5C–F). The results highlighted alterations in primary functional annotations, particularly biotin biosynthesis and tRNA processing, which exhibited notably high expression levels in the D group. Regarding fungal functions, octane oxidation showed improvement in the C and D groups, while sulfate reduction I displayed elevated activity in the A, B, and C groups but decreased in the E group.

### 3.6. Identification of Metabolites and Functional Annotation of DEMs

This study identified a total of 5726 metabolites in the positive ion mode and 6045 metabolites in the negative ion mode, all passing the quality control using relative standard deviation values (Figure S4). A total of 262 DEMs were detected and identified among the groups. Among these, the largest number of differential metabolites was found in the comparison A vs. D, with 31 up-regulated and 25 down-regulated metabolites (Figure S5). The next highest number of differential metabolites was observed in groups A and E, with 32 up-regulated and 18 down-regulated metabolites.

**Figure 6:**
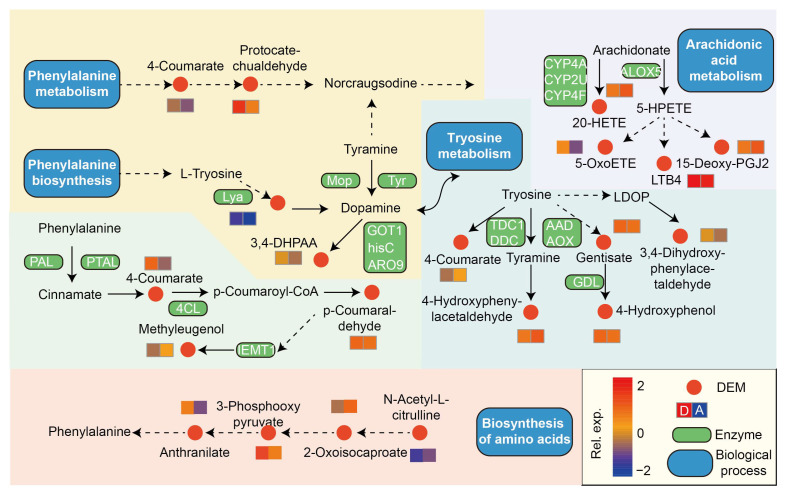
KEGG pathway analysis of differentially expressed metabolites using summarized KO genes and KEGG pathway modules. Red dots represent DEMs, while the heatmaps illustrate the relative abundance of DEMs related to identified metabolic pathways. Solid arrows denote direct interactions between two DEMs, while dashed lines signify unshown KO modules in the pathways.

The focused investigation on the D group, characterized by its exceptional nutrient dynamics and microbial diversity, provides valuable insights into the intricate metabolic pathways that underpin its ecological significance. Notably, in the A vs. D comparison, several pathways exhibited significant alterations, including “phenylpropanoid biosynthesis,” “tyrosine metabolism,” “arachidonic acid metabolism,” and “isoquinoline alkaloid biosynthesis” (Figure 6; Figure S6). Within the context of tyrosine metabolism, the study revealed changes in three up-regulated metabolites, specifically hydroquinone, gentisic acid, and 3,4-Dihydroxyphenylacetaldehyde, as well as two down-regulated metabolites, 4-hydroxycinnamic acid and 4-hydroxyphenylacetaldehyde, impacting the tyrosine metabolic process. Furthermore, isoquinoline alkaloid biosynthesis was influenced by a total of four DEMs, featuring two up-regulated and two down-regulated metabolites. In addition, the D group exerted a significant impact on phenylpropanoid biosynthesis by altering the expression levels of 4-hydroxycinnamic acid, (E)-3-(4-Hydroxyphenyl)-2-propenal, and methyleugenol. Arachidonic acid metabolism was also affected, with changes in four metabolites, including 12-Keto-tetrahydro-leukotriene B4, 15-Deoxy-d-12,14-PGJ2, 5-KETE, and 20-HETE. Interestingly, these four pathways were identified in the D vs. E group comparison, and an additional amino acid pathway, biosynthesis of amino acids, was characterized (Figure 6; Figure S6). This pathway demonstrated changes in metabolites such as 2-aminobenzoic acid, ketoleucine, phosphohydroxypyruvic acid, and N-a-Acetylcitrulline.

### 3.7. Interactions Among Soil Properties, Microbial Communities, and Soil Metabolic Multifunctionality

A correlation analysis was conducted to assess the relationships between α-diversity indices of soil bacterial communities, significant differential species at various taxonomic levels, and soil physicochemical factors (Table 1). The Chao1 richness index of bacterial communities did not exhibit significant correlations with any of the measured soil physicochemical factors (*p* > 0.05). The Shannon diversity index of bacterial communities showed significant positive correlations with AN, AK, and TN content (*p* < 0.05). The Pielou evenness index of bacterial communities displayed significant positive correlations with OC, AN, AK, and TN content (*p* < 0.05). Among the differential bacterial phyla, the relative abundance of Gemmatimonadetes exhibited significant positive correlations with soil OC (*p* < 0.05), while Firmicutes showed significant negative correlations with soil TN content (*p* < 0.05). Additionally, Rokubacteria displayed significant positive correlations with soil AK and TN content (*p* < 0.05). Similarly, the Chao1 richness index, Shannon diversity index, Pielou evenness index of fungal communities did not display significant correlations with any of the measured soil physicochemical factors (*p* > 0.05). Among the differential fungal phyla, the relative abundance of Ascomycota exhibited highly significant negative correlations with soil OC content (r = −0.967, *p* < 0.01) and highly significant negative correlations with soil AN content (r = −0.985, *p* < 0.01). It also showed significant negative correlations with soil TN content (*p* < 0.05). Calcarisporiellomycota exhibited significant negative correlations with soil TN content (*p* < 0.05). Mortierellomycota displayed a significant positive correlation with soil pH (*p* < 0.05), while Olpidiomycota exhibited a highly significant positive correlation with soil pH (r = 0.963, *p* < 0.01). To explore potential microbe-metabolite relationships, we performed RDA, which revealed clear associations between specific microbial genera and key metabolites (Figure S7). For instance, *Alicyclobacillus* and *Rokubacteriales* aligned strongly with metabolites such as kynurenine and X5-KETE in the bacterial dataset, while fungal genera like *Staphylotrichum* and *Ruhlandiella* correlated with X9-S-HPOD and X-E-3-4-Hydroxyphenyl-2-propenal.

**Figure 7:**
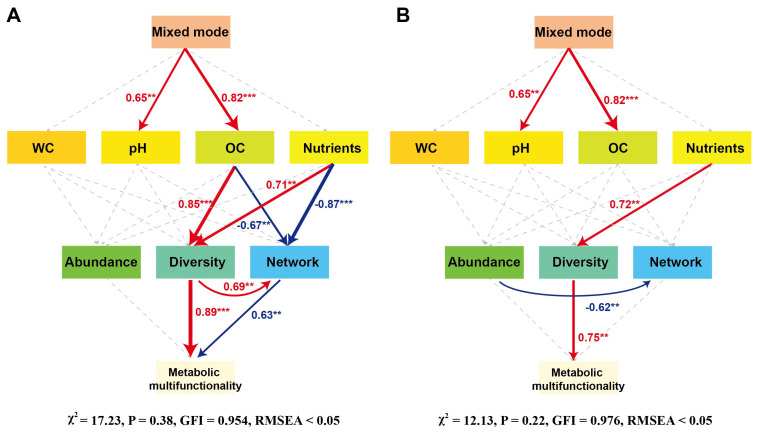
Structural equation model estimating the effects of soil properties, bacterial (**A**) and fungal (**B**) communities on soil metabolic multifunctionality in mixed forest patterns. Arrow width represents the strength of significant standardized path coefficients (*p* < 0.05), with red indicating positive correlation, and blue indicating negative correlation. Pathways with non-significant coefficients are represented by gray lines. *** *p* < 0.001; ** *p* < 0.01.

Subsequently, we conducted SEM analysis to demonstrate that mixed modes, OC, nutrients, and bacterial and fungal α-diversity were the primary influencing factors on soil metabolic multifunctionality, exerting greater impacts than soil pH, WC, and microbial abundance (Figure 7). Bacterial and fungal diversities had a direct and positive influence on soil metabolic multifunctionality, with standardized path coefficients of 0.89 and 0.75 (*p* < 0.01), respectively. Additionally, soil nutrients indirectly influenced soil microbial diversity, with standardized path coefficients of 0.71 for bacteria and 0.72 for fungi.

## 4. Discussion

### 4.1. Effects of Tree Species Composition on Soil Physicochemical Properties

Our study’s findings elucidate the significant impact of tree species composition on soil properties within mixed forest ecosystems, aligning with our objective to understand how different afforestation patterns influence soil ecology. Numerous studies indicate that establishing mixed forests can improve soil physicochemical properties to some extent and slow down the degradation of forest land in artificial forests [21,22]. During the development of artificial forests, the accumulation of understory litter increases, and its decomposition produces a large amount of acidic substances [23]. Notably, the higher soil water content observed in groups C and D, which have a higher proportion of *Cupressus*, suggests that the water retention capabilities of *Cupressus* are superior to those of *Eucalyptus*. This could be attributed to the physiological or morphological traits of *Cupressus*, such as root depth and leaf structure, which may enhance water retention and reduce evaporation rates [24]. Additionally, the variation in soil pH across the plots, with the highest values in the *Cupressus*-dominated group E, highlights how tree-specific litter and root exudates can modify soil chemical properties, potentially influencing microbial communities and plant nutrient uptake [25]. For instance, alkaline soils, as seen in group E, are known to impact the solubility of phosphates and micronutrients, which can affect plant growth and microbial activity [26]. However, this interpretation remains speculative in the absence of direct chemical characterization of litter or analysis of root exudate composition. Future studies incorporating detailed biochemical profiling of litter and root exudates would help confirm the specific compounds responsible for the observed pH variations. The significant differences in organic carbon (OC) and nitrogen (TN and AN) content, especially higher in group D, indicate a more robust nutrient cycling under mixed forests with a predominant presence of *Cupressus*. This can be associated with the slow decomposition rates of *Cupressus* litter compared to *Eucalyptus*, contributing to a more sustained release of nutrients. Such findings are critical for forest management, suggesting that strategic tree species selection can optimize soil health and enhance ecological resilience. For example, in regions prone to drought or poor soil fertility, integrating *Cupressus* into mixed forests could be advantageous for improving soil moisture and nutrient status, thereby supporting more stable forest ecosystems [27]. Moreover, the marked differences in potassium and phosphorus dynamics across the groups further underline the complex interactions between tree species and soil mineral content, guiding targeted interventions in forest composition to achieve desired ecological outcomes.

### 4.2. Microbial Community Structure and Ecological Implications

Soil microorganisms play a dominant role in almost all soil ecological processes, such as nutrient mineralization and decomposition, significantly influencing the functionality of forest ecosystems and the sustainability of the soil under the forest [28]. Studies indicate that the mixed planting of tree species with different functional traits can also reduce specific pathogenic microorganisms, positively affecting the health of the ecosystem [29]. Chloroflexi can degrade chitin, cellulose, or hemicellulose in the soil, and its higher relative abundance is associated with the presence of more undecomposed organic matter in the soil. The increase in the abundance of Acidobacteria may indicate changes in the ecological functions of the soil due to environmental stress, serving as an indicator of soil degradation under intensive agriculture scenarios. For soil fungi, the unique fungal composition observed in Group D signifies a distinctive ecological niche shaped by the lower abundance of Ascomycota and a significantly higher prevalence of Basidiomycota compared to other restoration groups. Notably, this fungal profile in Group D is characterized by dominant genera, including *Penicillium*, *Talaromyces*, *Fusarium*, *Aspergillus*, *Hyphodontia*, *Staphylotrichum*, and *Mortieralla*. *Penicillium* and *Aspergillus* are well-known for their versatile metabolic capabilities, contributing to nutrient cycling and organic matter decomposition in soil ecosystems [30,31]. *Talaromyces*, with its diverse enzymatic repertoire, participates in the breakdown of complex organic compounds, influencing nutrient availability [32]. *Fusarium*, a common soil inhabitant, plays crucial roles in plant-pathogen interactions and nutrient mobilization [33]. The nuanced exploration of these dominant genera provides a glimpse into their ecological functions, such as nutrient cycling, organic matter decomposition, and potential interactions with plants and other microbes.

### 4.3. Functional Microbial Adaptations and Ecosystem Processes

Notably, the D group emerged as a hotspot of microbial diversity, boasting a richness of both bacterial and fungal taxa, exemplified by the identification of specific taxa such as *Acidothermus*, *Conexibacter*, and *AD3* among bacteria, and *Penicillium*, *Aspergillus*, *Chaetomium*, and *Oidiodendron* among fungi. In contrast, the E group exhibited a comparatively lower abundance of taxa. The functional annotation analysis provided critical insights into the microbial functions within the forest ecosystems, notably showcasing significant shifts in biotin biosynthesis and tRNA processing pathways, with particular emphasis on the D group. Furthermore, the accentuated modifications in tRNA processing pathways within the D group suggest variations in gene expression and the translation of genetic information into functional proteins, possibly influencing the microbial community’s functional potential [34]. These findings signify unique functional adaptations within the microbial communities of the D group, underscoring their distinct metabolic and genetic processing capacities, which may have implications for the overall ecosystem functionality and adaptability compared to other forest groups. Overall, these results illuminate the intricate relationship between microbial taxonomy and functionality, providing a foundation for comprehending the underlying processes in soil ecosystems subjected to diverse restoration approaches.

### 4.4. Metabolomic Shifts and Microbe–Metabolite Interactions

Leveraging KEGG pathway analysis in a comparative context with the A and E groups unraveled substantial alterations in key pathways. For instance, the up-regulation of metabolites in the tyrosine metabolic process, such as hydroquinone, gentisic acid, and 3,4-Dihydroxyphenylacetaldehyde, suggests an enhanced utilization or synthesis of these compounds, possibly driven by specific microbial activities responding to the nutrient-rich environment [35,36]. Similarly, alterations in pathways could be indicative of adaptations in response to the microbial community’s functional dynamics, influenced by the unique characteristics of the D group. The isoquinoline alkaloid biosynthesis pathway, affected by four differentially expressed metabolites, may signify intricate interactions between the microbial community and plant secondary metabolites, potentially linked to ecological roles or defense mechanisms [37,38]. The positive correlation between (E)-3-(4-Hydroxyphenyl)-2-propenal and the *Candidatus_Koribacter* suggests a potentially symbiotic relationship. Conversely, the contrasting correlations of 4-Hydroxycinnamic acid with the *Alicyclobacillus* and its associations with *Rokubacteriales*, *KD4*-*96*, and *Rubrobacter* hint at differential metabolic responses within the bacterial community. These microbial-metabolite associations echo findings from similar studies, emphasizing the pivotal role of microbial community dynamics in shaping soil ecology.

To enhance our understanding of the intricate dynamics within forest ecosystems, several avenues for future research merit attention. The current study, while providing valuable insights into microbial communities and metabolic pathways in *Eucalyptus*–*Cupressus* mixed forests, is limited by its single time-point design and restricted geographic scope, which may not fully capture seasonal or site-specific variability. Longitudinal studies tracking temporal changes across seasons and years could reveal how soil microbial and metabolic profiles shift over time, offering a more comprehensive ecological perspective. Additionally, while our correlation analyses between microbial taxa and metabolites revealed meaningful associations, they remain correlational rather than causal. Future research should incorporate mechanistic approaches, such as metagenomics, metatranscriptomics, or stable isotope probing, to unravel the specific functional roles of microbial taxa and validate their contributions to soil biochemical processes in mixed forest systems. From a management perspective, our findings suggest that incorporating *Cupressus* into *Eucalyptus* plantations, particularly at a 1:2 ratio, can improve soil moisture, organic carbon, and nitrogen levels while enhancing microbial diversity and functional potential. This strategy may help mitigate common issues associated with monoculture plantations, such as nutrient depletion and poor soil structure, offering a nature-based solution for building more resilient and ecologically sustainable forest ecosystems in subtropical regions.

## 5. Conclusions

This study demonstrates that mixing *Eucalyptus* with *Cupressus*, particularly at a 1:2 ratio, can significantly improve soil conditions and promote a more diverse and functionally enriched soil microbiome. The observed increases in organic carbon, nitrogen, and water content, along with shifts in dominant microbial taxa and enriched metabolic pathways, indicate that species composition plays a central role in shaping soil ecological function. While our findings are limited to a single site and time point, they provide clear evidence that incorporating *Cupressus* into *Eucalyptus* plantations may help mitigate issues commonly associated with monoculture plantations, such as soil nutrient depletion, reduced microbial diversity, and poor moisture retention. These results support the application of mixed-species afforestation as a practical strategy to enhance soil health and sustainability in subtropical artificial forest systems. Future work should expand on these findings through multi-season, multi-site studies and deeper functional validation using metagenomics and metabolomics.

## Figures and Tables

**Table 1:** Pearson correlation coefficients between bacterial (16S) and fungal (ITS) community α-diversity indexes, dominant phyla, and soil physicochemical properties.

		Indexes	WC	pH	OC	AN	AP	AK	TN	TP	TK
16S	α-diversity	Chao1	0.428	0.508	0.769	0.849	0.216	0.775	0.856	−0.285	0.142
		Shannon	0.23	0.583	0.865	0.886 *	0.243	0.904 *	0.938 *	−0.178	0.223
		Pielou	0.063	0.549	0.917 *	0.885 *	0.18	0.945 *	0.948 *	−0.058	0.308
	Phylum	Firmicutes	−0.104	−0.765	−0.741	−0.777	−0.551	−0.872	−0.899 *	0.039	−0.293
		Gemmatimonadetes	0.173	0.278	0.902 *	0.868	−0.124	0.815	0.843	−0.112	0.267
		Acidobacteria	0.057	0.402	−0.273	−0.162	0.648	−0.086	−0.052	0.022	−0.028
		Verrucomicrobia	0.398	−0.47	0.005	−0.006	−0.748	−0.125	−0.148	−0.62	−0.532
		Rokubacteria	0.046	0.762	0.811	0.812	0.477	0.946 *	0.944 *	−0.064	0.272
		Chloroflexi	−0.476	−0.48	−0.734	−0.825	−0.192	−0.737	−0.822	0.335	−0.093
		Actinobacteria	0.527	0.398	0.757	0.856	0.112	0.707	0.815	−0.316	0.132
ITS	α-diversity	Chao1	0.348	−0.444	−0.604	−0.457	−0.098	−0.739	−0.606	−0.006	−0.124
		Shannon	0.35	0.612	0.098	0.217	0.549	0.34	0.346	−0.479	−0.307
		Pielou	0.17	0.788	0.315	0.368	0.598	0.619	0.564	−0.45	−0.252
	Phylum	Ascomycota	−0.192	−0.229	−0.967 **	−0.985 **	0.006	−0.755	−0.892 *	−0.264	−0.68
		Basidiomycota	0.568	−0.349	0.714	0.769	−0.629	0.329	0.502	−0.11	0.298
		Calcarisporiellomycota	−0.151	−0.643	−0.834	−0.877	−0.436	−0.861	−0.935 *	−0.075	−0.45
		Chytridiomycota	0.106	0.352	−0.28	−0.159	0.602	−0.118	−0.07	0.006	−0.034
		Mortierellomycota	−0.76	0.916 *	0.274	0.149	0.918	0.646	0.489	0.458	0.368
		Olpidiomycota	−0.463	0.963 **	0.338	0.293	0.959	0.683	0.592	0.288	0.316
		Rozellomycota	−0.852	0.826	0.313	0.149	0.8	0.647	0.475	0.535	0.424
		Zoopagomycota	0.331	−0.466	−0.361	−0.221	−0.14	−0.598	−0.417	0.208	0.172

Note: */** indicates significant values (*p* < 0.05 or 0.01). Abbreviations: WC, water content; pH, soil pH; OC, organic carbon; AN, available nitrogen; AP, available phosphorus; AK, available potassium; TN, total nitrogen; TP, total phosphorus; TK, total potassium.

## Data Availability

The sequencing data were submitted to the Sequence Read Archive (SRA) database with BioProject accession number PRJNA1043821 and MTBLS9022. Other data used to support the findings are available from the corresponding author upon request.

## Supplementary Materials

The following supporting information can be downloaded at: https://www.mdpi.com/article/10.3390/biology14121667/s1.
