# Supplementary material for: Improving Soil Properties and Microbiomes by Mixed Eucalyptus–Cupressus Afforestation
Source: Biology (Basel). 2025 Nov 24;14(12):1667. doi: 10.3390/biology14121667 (PMC12729669; doi:10.3390/biology14121667)
Supplement: Supplementary file 1 [file biology-14-01667-s001.zip › Supplementary Figure S1-S7.pdf]

Supplementary Materials

Improving Soil Properties and Microbiomes by Mixed *Eucalyptus–Cupressus* Afforestation

You-Wei Zuo [1,2], Yu-Ying Liu [1,2], Ya-Xin Jiang [3], Wen-Qiao Li [1,2], Yang Peng [1,2], Sheng-Mao Zhou [1,2], Shi-Qi You [1,2], Sheng-Qiao Liu [1,2] and Hong-Ping Deng [1,2,*]

[1] Center for Biodiversity Conservation and Utilization, Key Laboratory of Eco-Environment in the Three Gorges Reservoir Region, Ministry of Education, School of Life Sciences, Southwest University, Beibei, Chongqing 400715, China; youweiz@swu.edu.cn (Y.-W.Z.); ying91ly@163.com (Y.-Y.L.); liwenqiao126@email.swu.edu.cn (W.-Q.L.); 15182375576@139.com (Y.P.); lareinazhou99@email.swu.edu.cn (S.-M.Z.); 18702306241@163.com (S.-Q.Y.); l20210709@swu.edu.cn (S.-Q.L.)

[2] Chongqing Key Laboratory of Plant Resource Conservation and Germplasm Innovation, Institute of Resources Botany, School of Life Sciences, Southwest University, Beibei, Chongqing 400715, China

[3] College of Pharmaceutical Sciences, Southwest University, Beibei, Chongqing 400715, China; 13193009196@163.com

* Correspondence: denghp@swu.edu.cn; Tel.: +86-13883395687

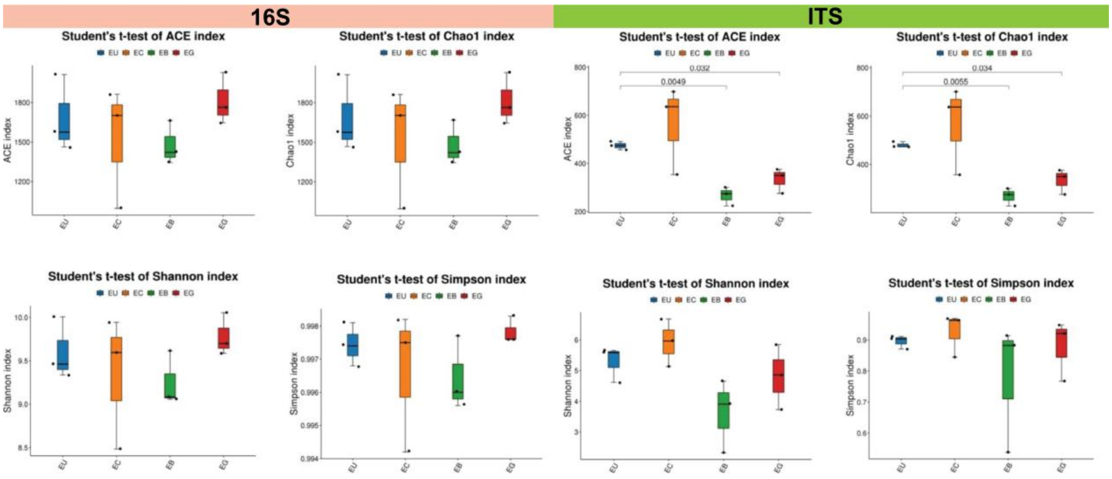

Figure S1. Box plots of soil bacterial and fungal alpha diversity indices in different mixed types of plantation. EC: *Eucalypt-Cypress* mixed forest; EB: *Eucalypt-Ficus* mixed forest; EG: *Eucalypt-Ginkgo* mixed forest; EU: *Eucalypt* pure fores.

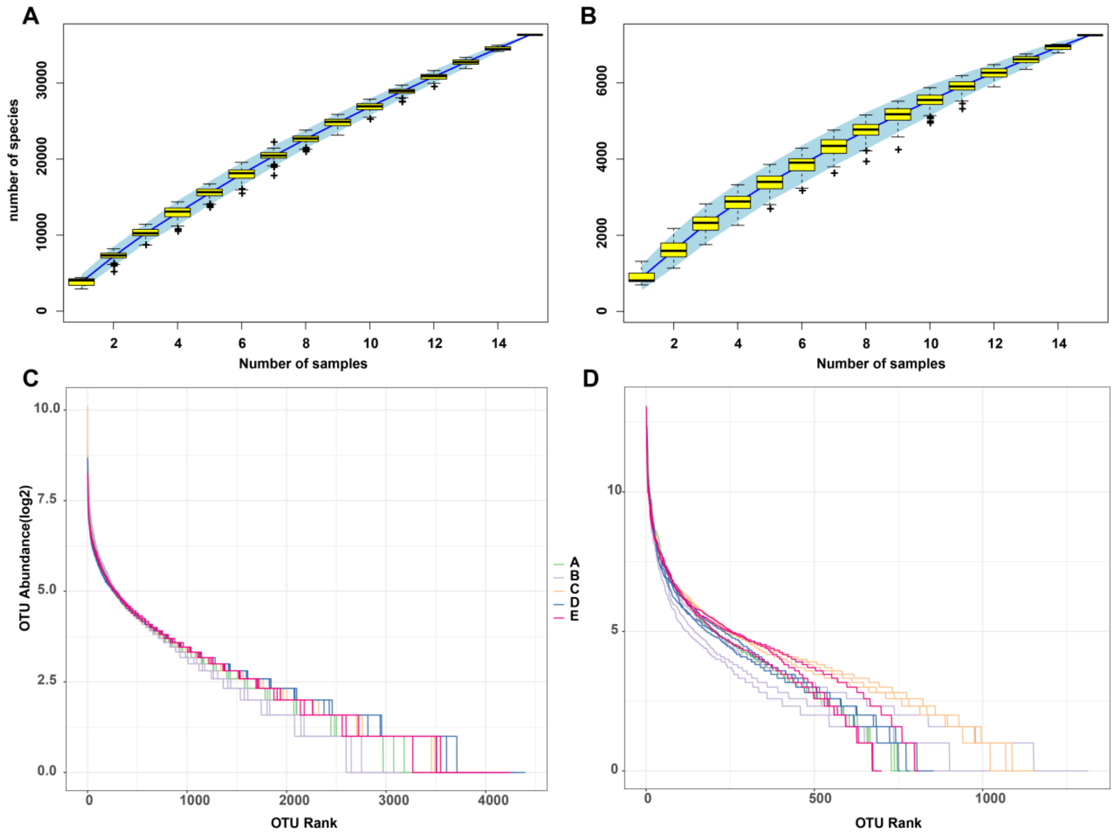

Figure S2. (A & B) Species accumulation curve in bacterial and fungal communities. The x-axis denotes the sample size, while the y-axis represents the observed number of species, with the blue shadow indicating the curve's confidence interval. This illustrates the rate at which new species are observed as the sample size grows during the population sampling process. (C & D) Abundance grade plot in bacterial and fungal communities. Each line represents a sample (group), with its length on the horizontal axis depicting the number OTU in that particular sample. The line's flatness reflects the community's composition uniformity: flatter lines indicate smaller differences in abundance within the community, signifying higher uniformity, while steeper lines indicate lower evenness due to greater abundance differences.

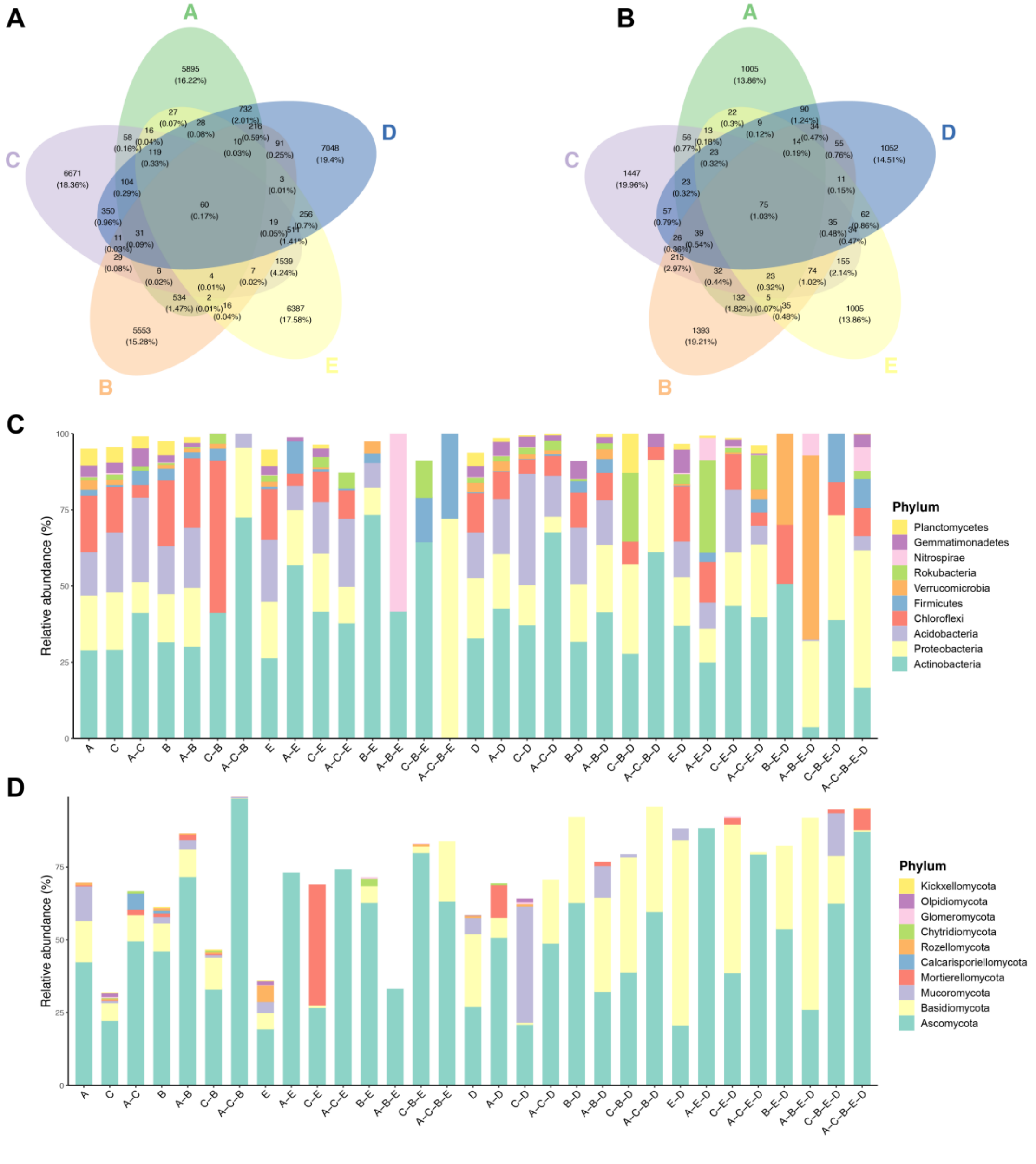

Figure S3. (A & B) Bacterial and Fungal Sample OTU Venn Diagram. (C & D) Histogram of the number of OTUs in different phylum of the Bacterial and Fungal Venn diagrams.

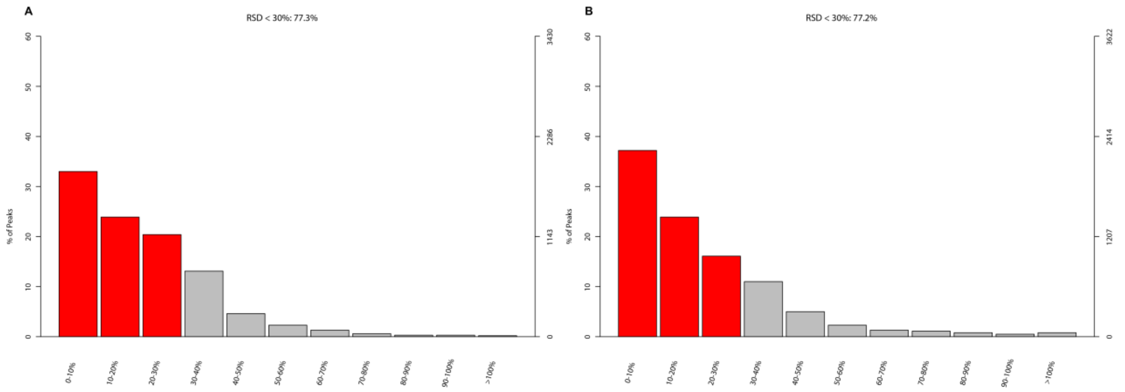

Figure S4. (A & B) Distribution maps of relative standard deviation (RSD) for positive and negative ions. In the quality control (QC) samples, when RSD < 30%, approximately 65% of characteristic peaks reached this ratio, signifying good data quality. The left vertical axis represents the proportion, the right vertical axis represents the specific quantity, and the horizontal axis denotes the range of RSD values.

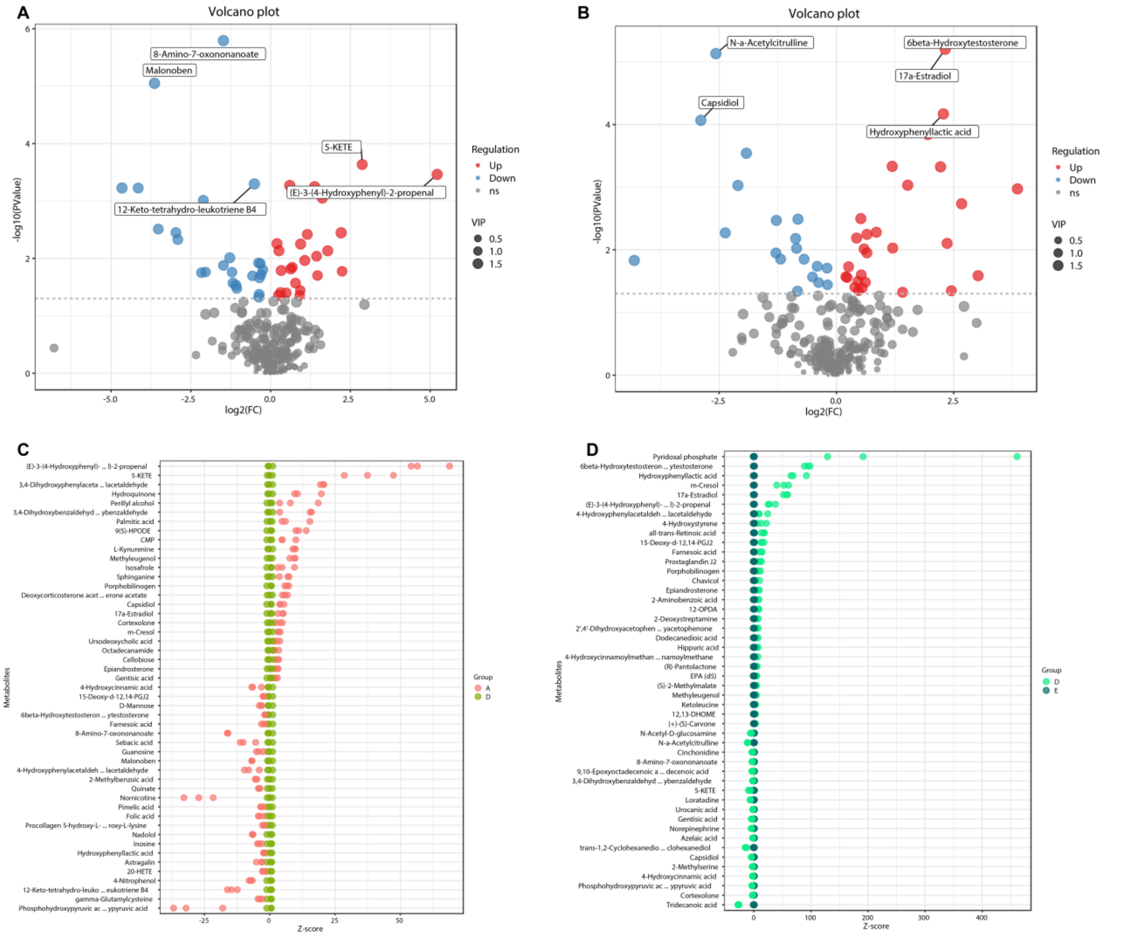

Figure S5. (A & B) The volcano plots showcase variances in metabolites between the A vs D and D vs E comparison groups. Each data point in the graph illustrates a specific metabolite, with the horizontal axis indicating the Log2 log-scale quantitative variance between the two samples. The vertical axis represents the -log10 pair of values indicating the p-value. (C & D) Depict the Z-score plot for differentially expressed metabolites.

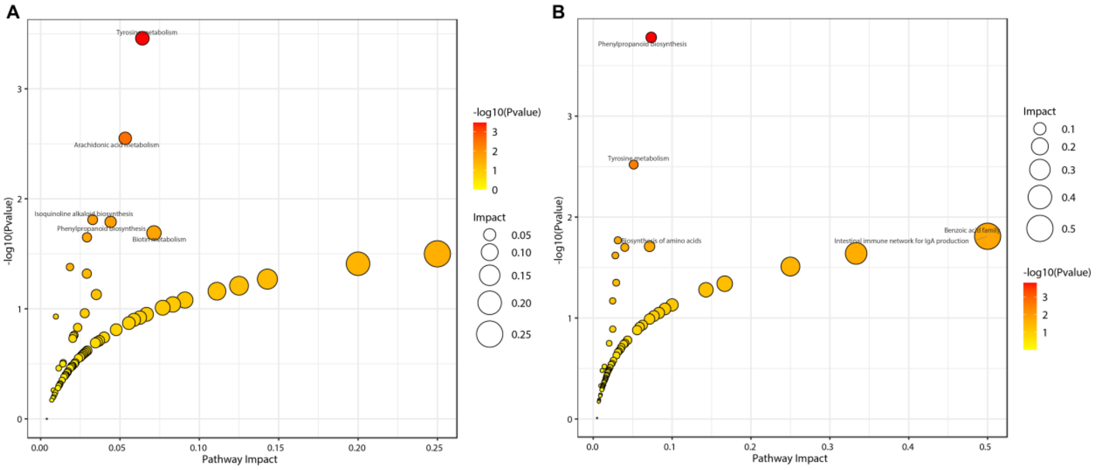

Figure S6. (A & B) Bubble maps illustrate the metabolic pathway influencers in the A vs D and D vs E comparison groups. Each point denotes a specific metabolic pathway, with the horizontal axis indicating the Impact value enriched into different metabolic pathways. The vertical axis shows the -log10 (P) value derived from the hypergeometric distribution test, where a lower P-value denotes the significance of the detected differential metabolites within the pathway.

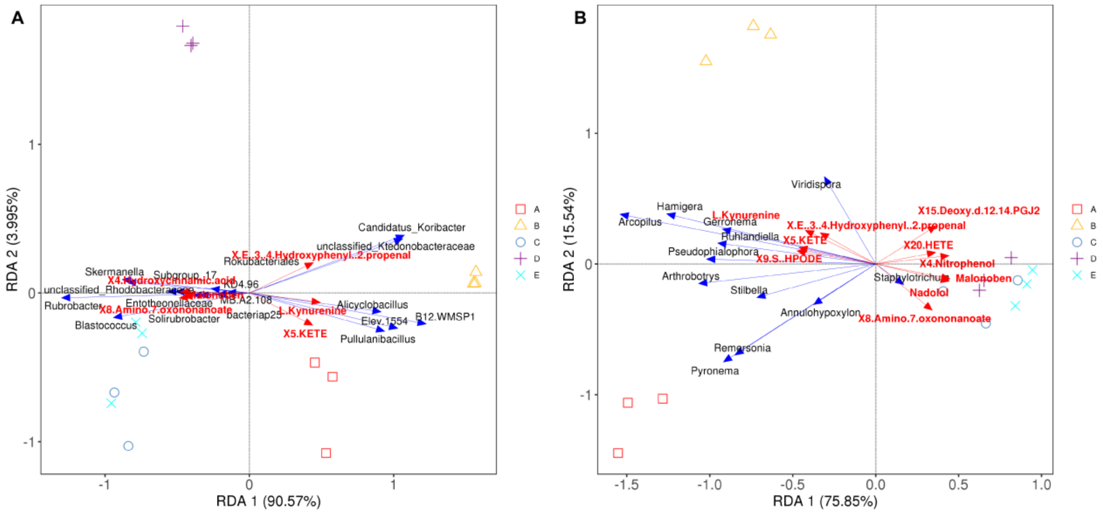

Figure S7. Redundancy analysis (RDA) of microbial taxa and metabolite profiles.

(A) RDA plot showing associations between bacterial community structure and metabolite composition. (B) RDA plot showing associations between fungal community structure and metabolite composition.
